# Genome-Wide Identification and Characterization of Chemosensory Gene Families in the Mayfly *Parafronurus youi* (Ephemeroptera: Heptageniidae)

**DOI:** 10.3390/genes17050549

**Published:** 2026-05-04

**Authors:** Haixin Li, Jinfeng Li, Qi Zhang, Muyang Li, Chao Xue, Ran Li

**Affiliations:** School of Life Sciences, Qufu Normal University, Qufu 273165, China

**Keywords:** Ephemeroptera, mayfly, *Parafronurus youi*, chemosensory genes, genome-wide identification

## Abstract

**Background:** Chemoreception plays a central role in how insects perceive environmental chemical cues and regulate essential behaviors. Mayflies (Ephemeroptera), among the earliest-diverging lineages of winged insects, are important bioindicators of freshwater ecosystems, yet their chemosensory gene repertoire remains poorly characterized. **Methods**: Using the high-quality genome of *Parafronurus youi*, we conducted a genome-wide identification and comparative analysis of six major chemosensory gene families. **Results**: We identified 72 candidate chemosensory genes, including 10 OBPs, 15 CSPs, 9 ORs, 14 GRs, 23 IRs, and 1 SNMP. These gene families differed markedly in physicochemical properties, conserved motifs, domain architecture, gene structure, chromosomal distribution, and phylogenetic relationships. Chemosensory genes were unevenly distributed across the 11 chromosomes, with several families showing clustered organization, whereas no obvious collinear relationships were detected. Compared with representative terrestrial insects, *P. youi* possesses a comparatively compact chemosensory repertoire, although this comparison should be interpreted cautiously because data sources and annotation strategies differ among studies. Different gene families showed distinct evolutionary patterns, including structurally conserved soluble binding proteins and co-receptors, limited diversification in several receptor families, and a comparatively well-represented IR repertoire. **Conclusions**: This study provides the first genome-wide overview of chemosensory genes in Ephemeroptera and offers a basis for future functional and evolutionary studies of chemoreception in palaeopteran insects.

## 1. Introduction

Chemoreception is a fundamental biological process through which organisms detect and interpret chemical cues from their environment. In insects, the olfactory system mediates the perception of diverse volatile compounds and underlies key behaviors such as food searching, mate recognition, and predator avoidance. By contrast, the gustatory system primarily detects water-soluble stimuli and plays central roles in feeding regulation and reproduction-related responses [1,2]. The insect chemosensory system comprises several classes of specialized proteins, including odorant-binding proteins (OBPs), chemosensory proteins (CSPs), odorant receptors (ORs), gustatory receptors (GRs), ionotropic receptors (IRs), and sensory neuron membrane proteins (SNMPs) [3,4,5].

Among these components, OBPs and CSPs act as carrier proteins that transport pheromones and environmental odorants during insect chemoreception [6]. Both families are typically water-soluble, low-molecular-weight proteins, with molecular masses of approximately 13–17 kDa and polypeptide lengths of about 120–170 amino acids [7,8,9]. OBPs generally contain six highly conserved cysteine residues that form three disulfide bonds, thereby stabilizing their compact tertiary structure and contributing to their evolutionary conservation [3,7]. According to the number and arrangement of cysteine residues, OBPs can be classified into five subtypes: Classic, Minus-C, Plus-C, Dimer, and Atypical OBPs [10,11]. CSPs, by contrast, contain four conserved cysteine residues that form two disulfide bonds [12]. Compared with OBPs, CSPs are often considered more conserved, and amino acid identity between CSPs from different insect species can reach approximately 50% [13,14]. Notably, CSPs show no substantial sequence similarity to OBPs, suggesting that the two protein families evolved independently [13,14].

Insect chemosensory receptors mainly comprise four major families: ORs, GRs, IRs, and SNMPs. These receptors convert extracellular chemical stimuli into electrical signals that are subsequently transmitted to sensory neurons [4,15]. ORs and GRs generally encode proteins of 350–500 amino acids and typically contain seven hydrophobic transmembrane domains (TMDs) [16]. ORs function as odor-gated ion channels composed of odorant-specific OR subunits and the highly conserved odorant receptor co-receptor (ORco), which together form functional receptor complexes [17,18,19]. ORco is highly conserved across insect orders and is essential for receptor trafficking, membrane localization, and signal transduction [20]. Evolutionary studies have suggested that ORs originated from the GR family [21]. GRs were first identified in *Drosophila melanogaster* [22] and have subsequently been characterized in numerous insects, including representatives of Diptera [23,24], Lepidoptera [25], and Coleoptera [26]. They are primarily expressed in gustatory receptor neurons located in taste organs and can be divided into four major subfamilies according to ligand specificity: fructose receptors, sugar receptors (excluding fructose receptors), carbon dioxide (CO_2_) receptors, and bitter receptors [25,27].

Ionotropic receptors (IRs) are ligand-gated ion channels derived from ionotropic glutamate receptors (iGluRs). They typically encode proteins of 600–1000 amino acids and generally contain three TMDs [16,28]. IRs are commonly divided into two groups: conserved antennal IRs and species-specific divergent IRs [29]. Antennal IRs are mainly expressed in antennae and localized in coeloconic sensilla neurons, where they mediate the detection of acids, amines, and amino acids, whereas divergent IRs are more broadly expressed in other tissues [30]. Their function depends on co-expression with conserved co-receptors, notably IR8a, IR25a, IR76b, and IR93a [31]. SNMPs belong to the CD36 receptor family and are double-transmembrane proteins first identified in pheromone-sensitive olfactory sensory neurons of Lepidoptera [5]. In most insects, SNMPs are classified into two subfamilies, SNMP1 and SNMP2, both of which are implicated in the binding and transport of hydrophobic ligands [32,33,34].

Ephemeroptera, commonly known as mayflies, represent one of the earliest-diverging lineages of winged insects [35]. They are highly sensitive to water quality and are therefore widely recognized as important bioindicators of freshwater ecosystem health [36]. This ecological sensitivity may be closely linked to their ability to perceive environmental chemical cues through the chemosensory system. However, research on Ephemeroptera and the broader Palaeoptera lineage (Ephemeroptera and Odonata) has long focused on morphology, taxonomy, ecology, and life history, whereas molecular investigations remain relatively limited [37,38,39,40,41,42]. In particular, systematic studies of chemosensory genes in mayflies are still lacking, leaving the molecular basis of their chemoreception and ecological sensitivity poorly understood.

In this study, we conducted a genome-wide identification and systematic analysis of six major chemosensory gene families in *P. youi* based on a recently published high-quality genome assembly [43]. In total, 72 candidate chemosensory genes were identified, including members of the OBP, CSP, OR, GR, IR, and SNMP families. We further performed physicochemical characterization, gene structure analysis, chromosomal localization, collinearity analysis, and phylogenetic reconstruction. The results indicate that chemosensory genes in *P. youi* are unevenly distributed across chromosomes and exhibit clear family-specific evolutionary patterns. Overall, the chemosensory repertoire of *P. youi* appears comparatively compact relative to those reported for representative terrestrial insects, although such comparisons should be interpreted with caution because they may be affected by differences in data sources and annotation strategies. These findings provide the first comprehensive insight into the chemosensory gene repertoire of a mayfly species and contribute to a better understanding of the evolution of chemosensory systems in palaeopteran insects. Furthermore, this study provides genomic resources for future functional and multi-omics studies on chemosensory mechanisms in mayflies.

## 2. Materials and Methods

### 2.1. Identification of Chemosensory Gene Families in P. youi

The whole-genome data of *P. youi* used in this study were obtained from our previously reported genome assembly and are publicly available in the Figshare database [43]. To identify candidate chemosensory genes, protein sequences of OBPs, CSPs, ORs, GRs, IRs, and SNMPs from representative insect species were retrieved from the National Center for Biotechnology Information (NCBI) database. These sequences were used as queries in homology-based searches against the *P. youi* genome using DIAMOND v2.0.15 with an E-value threshold of 1 × 10^−5^; top hits were retained based on the highest bit scores and adequate alignment coverage for subsequent analyses [44]. To improve identification accuracy, Hidden Markov Model (HMM) searches were also performed using HMMER v3.3.2 [45]. HMM profiles corresponding to chemosensory gene families were obtained from the Pfam database and used to scan the *P. youi* protein dataset with an E-value cutoff of 1 × 10^−5^. Candidate sequences supported by either homology-based or HMM-based searches were retained for further verification. 

To further verify the initially screened candidate sequences, reciprocal BLAST analyses were performed. Candidate chemosensory protein sequences from *P. youi* were used as queries in reciprocal BLAST searches against the NCBI non-redundant (nr) protein database and the original reference insect protein dataset, with an E-value threshold of 1 × 10^−5^. Sequences showing consistent best matches to known chemosensory genes were regarded as reliable candidates and retained for downstream analyses.

After sequence verification, redundant sequences, defined as those showing more than 90% overall nucleotide or amino acid sequence similarity and more than 85% full-length alignment coverage, were manually removed. Incomplete candidates, including sequences lacking complete ORFs with both start and stop codons or showing evidence of incomplete genomic annotation, were also excluded. Conserved domains of the remaining candidate proteins were then confirmed using the NCBI Conserved Domain Database (CDD) with default parameters [46], and sequences lacking characteristic chemosensory-related domains were excluded. After this curation, the retained sequences, irrespective of protein length, were regarded as members of the corresponding chemosensory gene families. The nucleotide and amino acid sequences of all identified chemosensory genes are provided in Tables S1 and S2.

### 2.2. Analysis of Physicochemical Properties of Chemosensory Genes

The open reading frames (ORFs) of candidate chemosensory genes were identified using the NCBI Open Reading Frame Finder (ORFfinder). The physicochemical properties of the encoded proteins, including molecular weight (MW), isoelectric point (pI), instability index (II), aliphatic index (AI), and grand average of hydropathicity (GRAVY), were calculated using the ExPASy ProtParam tool [47].

Signal peptides of odorant transport proteins (OBPs and CSPs) were predicted using SignalP 6.0 with default parameters [48]. TMDs of chemosensory receptors (ORs, GRs, IRs, and SNMPs) were predicted using TMHMM v2.0 [49]. Subcellular localization of all chemosensory-related proteins was predicted using WoLF PSORT with the default settings [50].

### 2.3. Analysis of Conserved Motifs, Domains, and Gene Structures

Conserved motifs in chemosensory-related proteins were identified using MEME Suite v5.5.0 [51]. The parameters were set as follows: maximum number of motifs, 10; motif width, 6–50 residues; all other parameters were kept at their default values. A neighbor-joining (NJ) phylogenetic tree was constructed in MEGA 12 using the Poisson substitution model, pairwise deletion, and 1000 bootstrap replicates [52]. NJ was used for within-species structural visualization, whereas ML was used for cross-species phylogenetic inference.

Conserved domains of chemosensory-related proteins were identified using the NCBI CDD. Gene structure information, including coding sequences (CDS) and untranslated regions (UTRs), was extracted from the genome annotation (GFF) file and visualized using TBtools v1.120 [53]. Phylogenetic trees, motif composition, conserved domains, and gene structures were integrated and displayed using TBtools v1.120.

### 2.4. Chromosomal Localization and Collinearity Analysis

Gene position information was extracted from the GFF file of *P. youi*. The MG2C online tool was used to visualize the chromosomal distribution of chemosensory genes across the 11 chromosomes [54]. Gene duplication events and intraspecific collinearity relationships were analyzed using MCScanX with default parameters [55]. Tandem duplication events were defined as adjacent homologous genes located within a 200 kb genomic interval on the same chromosome. Collinearity relationships were visualized using the Advanced Circos function implemented in TBtools v1.120 [56].

### 2.5. Phylogenetic Analysis

Phylogenetic analyses were conducted using the amino acid sequences of chemosensory genes from *P. youi* and homologous sequences from other insect species [57]. Comparison species were selected according to three criteria. First, selected taxa represented phylogenetically relevant insect lineages with available chemosensory gene data. Second, publicly available genome or sequence resources of sufficient quality were required to support reliable sequence comparison. Third, the chemosensory gene families of the selected species had been previously identified or sufficiently annotated, enabling meaningful interspecific comparison. Because evolutionary rates and functional diversification differ among chemosensory gene families, the final taxon set was adjusted for each family under this unified framework. Multiple sequence alignments were performed using MAFFT v7.505 implemented in PhyloSuite with the L-INS-i algorithm, which is suitable for sequences with conserved domains [58]. Poorly aligned regions were removed using TrimAl v1.4 with the “automated1” mode [59]. A maximum-likelihood (ML) phylogenetic tree was constructed using IQ-TREE v2.2.0 under the best-fit substitution model selected by ModelFinder [60]. Branch support was assessed using the ultrafast bootstrap method with 5000 replicates. The resulting phylogenetic trees were visualized and edited using FigTree v1.4.4 [61] and further refined using the Evolview online platform [62]. The species included in the phylogenetic analyses and their corresponding color assignments are provided in Table S3.

## 3. Results

### 3.1. Identification and Characterization of Putative OBPs

Ten putative odorant-binding protein genes (PyouOBP1–PyouOBP10) were identified from the *P. youi* genome. All of them contained complete ORFs and encoded proteins ranging from 135 aa (PyouOBP5) to 252 aa (PyouOBP10), with a mean length of 158 aa. Their predicted MWs varied between 15.00 and 27.53 kDa, while the theoretical pIs ranged from 4.69 to 9.50 (Table S4). In addition, the II, AI, and GRAVY values spanned 12.52–56.72, 65.91–94.14, and −0.597–0.036, respectively. All full-length PyouOBPs possessed an N-terminal signal peptide of 18–24 aa and were predicted to be extracellularly localized, consistent with the canonical role of OBPs as soluble carriers in the sensillar lymph (Table S4).

**Figure 1:**
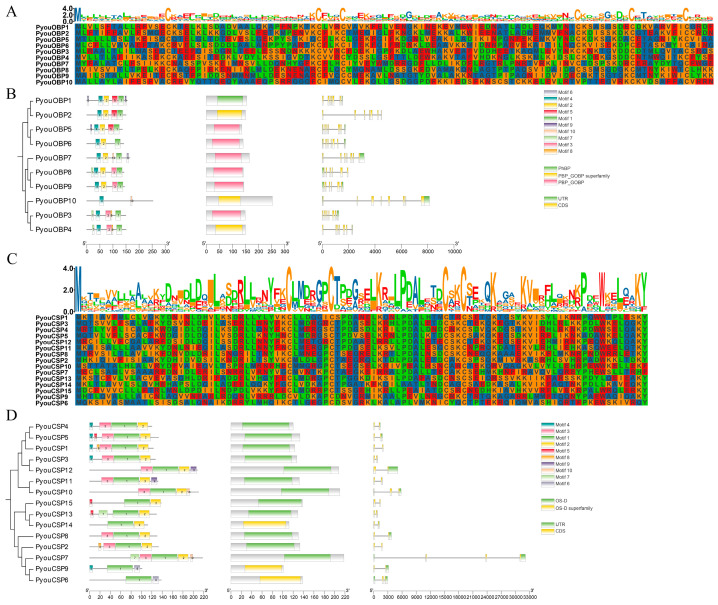
General characteristics of OBP and CSP genes and proteins in *P. youi*. Multiple sequence alignments of OBP (**A**) and CSP (**C**) proteins, and integrated analyses of phylogenetic relationships, conserved motifs, domains, and gene structures of OBP (**B**) and CSP (**D**) gene families in *P. youi*.

**Figure 2:**
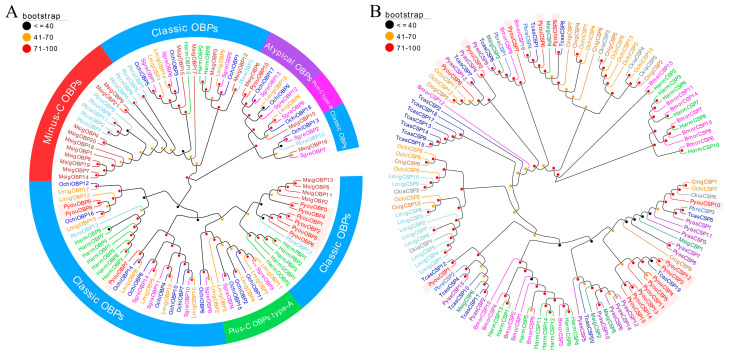
(**A**) Phylogenetic tree of putative OBPs from *P. youi* and other insects. A total of 108 sequences used for tree construction were obtained from seven species (10 from *P. youi*, 17 from *Locusta migratoria*, 18 from *Oxya chinensis*, 14 from *Schistocerca gregaria*, 15 from *Helicoverpa armigera*, 13 from *Protaetia brevitarsis*, and 21 from *Monolepta signata*). (**B**) Phylogenetic tree of putative CSPs from *P. youi* and other insects. A total of 127 sequences used for tree construction were obtained from 11 species (15 from *P. youi*, 18 from *Tribolium castaneum*, 4 from *P. brevitarsis*, 15 from *Pachyrhinus yasumatsui*, 6 from *M. signata*, 14 from *H. armigera*, 16 from *Bombyx mori*, 13 from *O. chinensis*, 10 from *L. migratoria*, 6 from *Ceracris kiangsu*, and 10 from *C. nigricornis*).

Sequence alignment further revealed that PyouOBP1–PyouOBP9 exhibited the six-cysteine signature typical of Classic OBPs, whereas PyouOBP10, which possesses two additional cysteine residues, was classified into the Atypical OBP subfamily (Figure 1A). This classification was further supported by phylogenetic reconstruction based on 108 OBP sequences from *P. youi* and six other insect species, which resolved the expected OBP lineages, including Classic, Atypical, Plus-C, and Minus-C groups (Figure 2A). The Plus-C lineage could be further subdivided into type-A and type-B according to the positions of the additional cysteine residues. Within this framework, all ten PyouOBPs were placed within the Classic or Atypical clades, consistent with their conserved cysteine patterns. Notably, PyouOBP7 showed pairwise 1:1 orthologous affinities with OchiOBP14 and LmigOBP8 among the three species, whereas PyouOBP10 formed a clear 1:1 orthologous relationship with MsigOBP6. Two closely associated paralog-like pairs, PyouOBP3/PyouOBP4 and PyouOBP8/PyouOBP9, were also evident in the phylogeny (Figure 2A).

### 3.2. Identification and Characterization of Putative CSPs

Fifteen candidate chemosensory protein genes were retrieved from the *P. youi* genome. Each gene contained a complete ORF, encoding proteins of 100 (PyouCSP9)–217 (PyouCSP7) aa in length, with an average of 142 aa (Table S5). The predicted MWs ranged from 11.37 to 24.24 kDa, and the theoretical pI values varied from 5.29 to 9.78. Their IIs ranged from 24.40 to 69.11, AIs from 69.47 to 98.65, and GRAVY values from −0.665 to 0.033. Signal peptide prediction indicated that 12 of the 15 CSPs harbored an N-terminal signal peptide of 17–23 aa, whereas PyouCSP7, PyouCSP10, and PyouCSP12 lacked a predicted signal peptide. Despite this difference, all members were predicted to be extracellular proteins. As shown in Figure 1C, every PyouCSP retained the characteristic four-cysteine motif of insect CSPs.

The evolutionary relationships of the CSP family were assessed using sequences from *P. youi* and 10 additional insect species. The resulting tree showed that several PyouCSPs maintained clear orthologous affinities with coleopteran homologs (Figure 2B). In particular, PyouCSP1, PyouCSP2, and PyouCSP9 each formed a 1:1 orthologous relationship with CSPs from other beetles. By contrast, PyouCSP3 and PyouCSP12, as well as PyouCSP4 and PyouCSP5, appeared as closely related sister pairs. A distinct cluster composed of PyouCSP13–PyouCSP15 was also recovered, representing an independent lineage within the tree (Figure 2B).

### 3.3. Identification and Characterization of Putative ORs

Nine odorant receptor genes were identified in *P. youi*, comprising one highly conserved co-receptor gene (PyouORco) and eight conventional OR genes (PyouOR1–PyouOR8). All ORs possessed complete ORFs. The encoded proteins varied markedly in size, ranging from 150 aa for PyouOR4 to 865 aa for PyouOR7, with an average length of 360 aa (Table S6). Their predicted MWs ranged from 16.66 to 99.34 kDa, and the theoretical pI values ranged from 5.01 to 9.12. The II, AI, and GRAVY values ranged from 29.17 to 46.27, 95.72 to 129.33, and 0.008 to 0.871, respectively. TMD prediction showed that PyouOR proteins contained 2–8 transmembrane helices, and subcellular localization analysis placed them predominantly in the plasma membrane, consistent with their expected role as membrane-bound olfactory receptors (Table S6).

**Figure 3:**
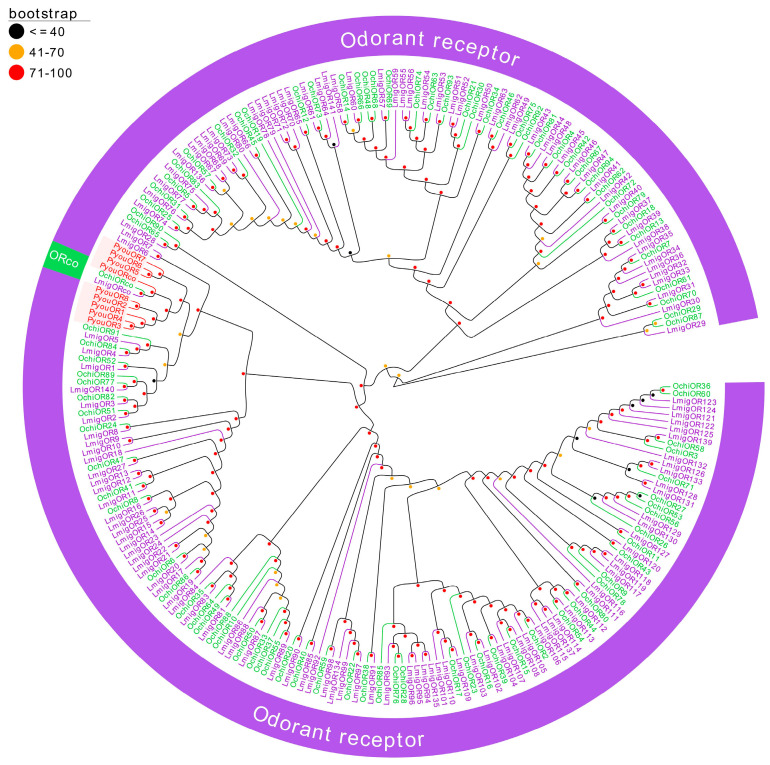
Phylogenetic tree of putative ORs from *P. youi* and other insects. A total of 245 sequences used for tree construction were obtained from three species (9 from *P. youi*, 142 from *L. migratoria*, and 94 from *O. chinensis*).

Phylogenetic analysis based on 245 OR sequences from *P. youi*, *L. migratoria*, and *O. chinensis* resolved the dataset into two principal lineages: the highly conserved ORco clade and the conventional OR clade (Figure 3). PyouORco clustered robustly with LmigORco and OchiORco, exhibiting pairwise 1:1 orthology among the three species and confirming the high conservation of this receptor across insect taxa. In contrast, the remaining PyouORs were distributed into two *P. youi*-specific clusters. One cluster comprised PyouOR1–PyouOR4 together with PyouOR8, whereas the other included PyouOR5–PyouOR7 (Figure 3).

### 3.4. Identification and Characterization of Putative GRs

Fourteen putative gustatory receptor genes (PyouGR1–PyouGR14) were identified from the *P. youi* genome. All of them contained complete ORFs and encoded proteins ranging from 341 aa (PyouGR9) to 511 aa (PyouGR1), with an average length of 408 aa (Table S7). The predicted MWs varied from 39.13 to 56.11 kDa, whereas the theoretical pIs covered a relatively broad range of 4.55–9.54. The IIs ranged from 33.72 to 55.72, the AIs from 89.95 to 122.18, and the GRAVY values from −0.448 to 0.703. Seven members, namely PyouGR1–PyouGR3, PyouGR6, PyouGR7, PyouGR10, and PyouGR12, were predicted to possess seven TMDs. Most PyouGRs were localized to the plasma membrane, which is consistent with their expected role in gustatory signal reception (Table S7).

**Figure 4:**
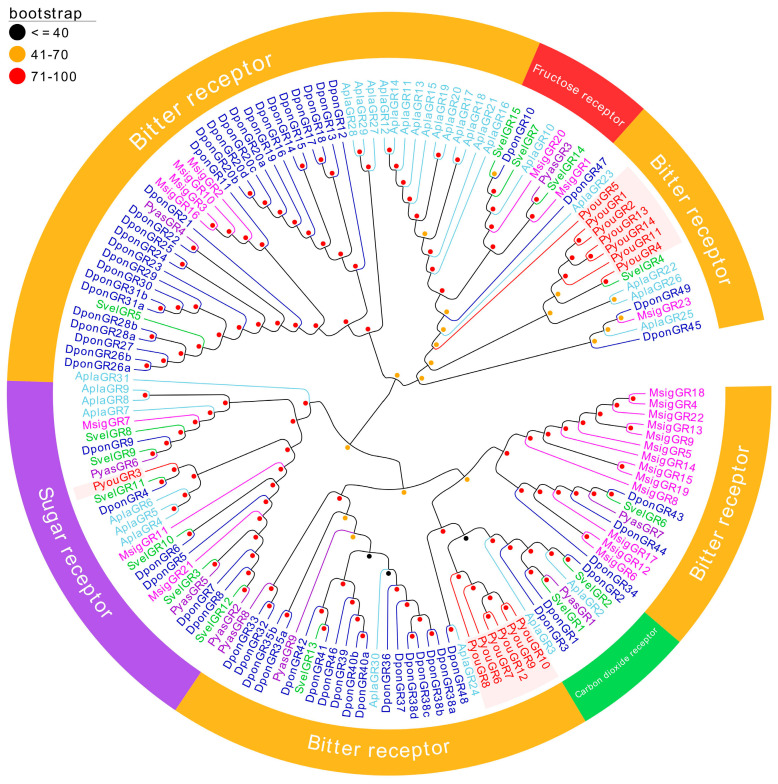
Phylogenetic tree of putative GRs from *P. youi* and other insects. A total of 151 sequences used for tree construction were obtained from six species (14 from *P. youi*, 9 from *P. yasumatsui*, 23 from *M. signata*, 60 from *Dendroctonus ponderosae*, 30 from *Agrilus planipennis*, and 15 from *Sympiezomias velatus*).

The phylogenetic tree inferred from 151 GR sequences representing six insect species indicated that the *P. youi* GR repertoire was restricted to two subfamilies (Figure 4). Thirteen members, including PyouGR1, PyouGR2, and PyouGR4–PyouGR14, were assigned to the bitter receptor lineage, whereas only PyouGR3 clustered within the sugar receptor lineage. No *P. youi* sequences were placed within the CO_2_ receptor or fructose receptor clades. Within the bitter receptor assemblage, the PyouGRs were further separated into two *P. youi*-enriched groups: one consisting of PyouGR6–PyouGR10 together with PyouGR12, and the other comprising PyouGR1, PyouGR2, PyouGR4, PyouGR5, and PyouGR11–PyouGR14. By contrast, PyouGR3 clustered within the sugar receptor lineage and showed pairwise 1:1 orthologous affinities with SvelGR11 and DponGR4 among the three species (Figure 4).

### 3.5. Identification and Characterization of Putative IRs and SNMPs

A total of 23 putative ionotropic receptor genes were identified in the *P. youi* genome, including 19 divergent IRs and four conserved co-receptors, namely PyouIR8a, PyouIR25a, PyouIR76b, and PyouIR93a (Table S8). All 23 genes possessed complete ORFs. The encoded proteins showed substantial variation in size, ranging from 283 aa (PyouIR5) to 1160 aa (PyouIR15), with an average length of 763 aa. The predicted MWs ranged from 31.95 to 128.32 kDa, and the pI values ranged from 5.45 to 10.13. Their IIs ranged from 33.03 to 56.32, AIs from 82.42 to 112.38, and GRAVY values from −0.257 to 0.167, with most members showing negative GRAVY values. Transmembrane analysis suggested that PyouIRs contained 1–4 TMDs, and subcellular localization prediction indicated that they were primarily membrane-associated (Table S8).

**Figure 5:**
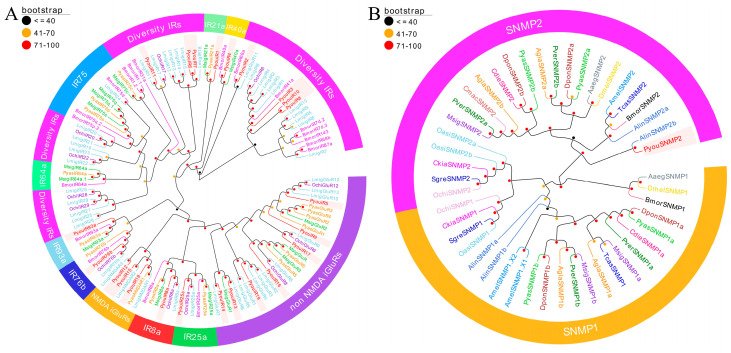
(**A**) Phylogenetic tree of putative IRs from *P. youi* and other insects. A total of 132 sequences used for tree construction were obtained from six species (23 from *P. youi*, 47 from *L. migratoria*, 12 from *O. chinensis*, 15 from *M. signata*, 16 from *P. yasumatsui*, and 19 from *B. mori*). (**B**) Phylogenetic tree of putative SNMPs from *P. youi* and other insects. A total of 47 sequences used for tree construction were obtained from 18 species (1 from *P. youi*, 3 from *M. signata*, 4 from *P. yasumatsui*, 2 from *O. chinensis*, 2 from *S. gregaria*, 3 from *Oedaleus asiaticus*, 2 from *C. kiangsu*, 2 from *Aedes aegypti*, 3 from *Apis mellifera*, 2 from *B. mori*, 2 from *D. melanogaster*, 2 from *T. castaneum*, 4 from *D. ponderosae*, 4 from *Anoplophora glabripennis*, 1 from *Callosobruchus maculatus*, 2 from *Curculio dieckamanni*, 4 from *Plagiodera versicolora*, and 4 from *Adelphocoris lineolatus*).

Phylogenetic reconstruction based on 132 IR sequences from six insect species placed the four co-receptors unambiguously into the conserved IR8a, IR25a, IR76b, and IR93a clades, respectively (Figure 5A). Among the remaining members, PyouIR12, PyouIR15, and PyouIR18 grouped within the NMDA iGluR lineage, whereas PyouIR5, PyouIR9, PyouIR13, PyouIR14, PyouIR16, PyouIR17, and PyouIR19 were assigned to the non-NMDA iGluR lineage. PyouIR1 and PyouIR4 clustered with the IR21a and IR40a clades, respectively. The other PyouIRs fell into the divergent IR subfamily. Unlike the OR and GR repertoires, the IR family did not show an obvious *P. youi*-specific clustered expansion in the phylogenetic tree (Figure 5A).

In addition to the IRs, only a single SNMP gene was detected in the *P. youi* genome and was designated PyouSNMP2 (Table S8). This gene encoded a 528-aa protein with a predicted MW of 58.04 kDa and a theoretical pI of 4.61. Its II, AI, and GRAVY values were 30.77, 101.34, and 0.069, respectively. PyouSNMP2 contained two TMDs and was predicted to localize to the plasma membrane, matching the typical structural properties of SNMPs (Table S8). Phylogenetic analysis of 47 SNMP sequences from 18 insect species resolved the family into the SNMP1 and SNMP2 subfamilies. PyouSNMP2 was placed within the SNMP2 clade and showed a 1:1 orthologous relationship with AsimSNMP2 (Figure 5B).

### 3.6. Conserved Motifs, Domains, and Gene Structures

**Figure 6:**
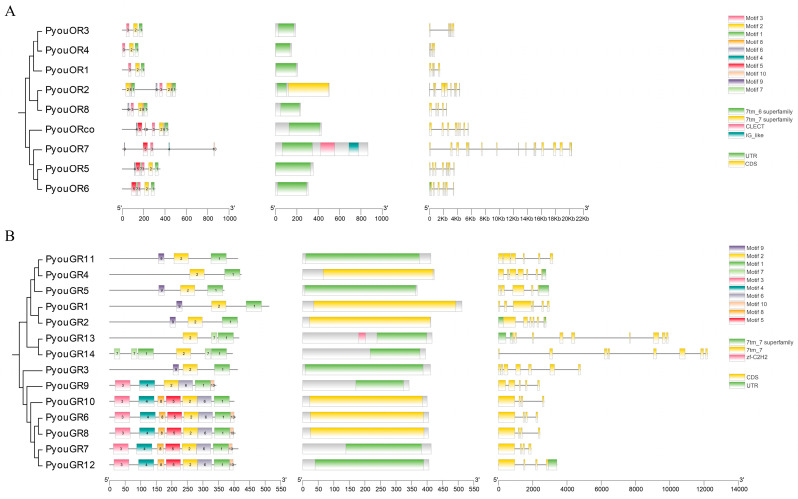
Phylogenetic relationships, conserved motifs, domains, and gene structures of OR (**A**) and GR (**B**) gene families in *P. youi*.

**Figure 7:**
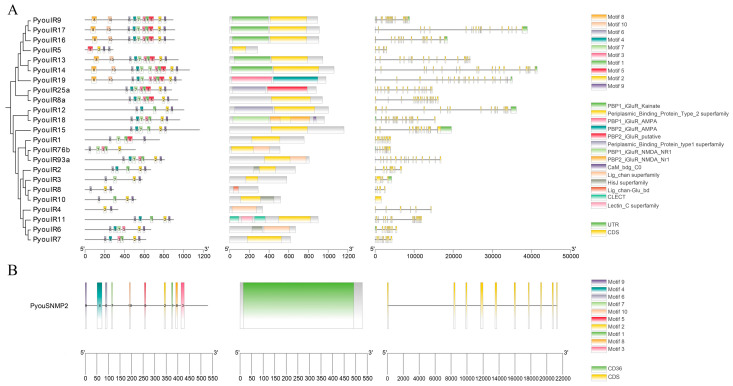
Phylogenetic relationships, conserved motifs, domains, and gene structures of IR (**A**) and SNMP (**B**) gene families in *P. youi*.

Integrated analyses of conserved motifs, domain composition, and exon–intron organization revealed clear structural differentiation among the chemosensory gene families (Figure 1, Figure 6 and Figure 7). Within the OBP family, motif 4 was retained in all members and represented the most conserved motif. All PyouOBPs possessed the characteristic PBP_GOBP/PhBP domain. Structurally, OBP genes contained 6–8 exons and 5–7 introns. Their genomic lengths showed moderate variation, with PyouOBP10 being the longest gene (8.1 kb), whereas 80% of the family members were shorter than 4 kb. Six OBP genes retained both 5′ and 3′ UTRs, while the remaining four contained only one annotated UTR (Figure 1B).

Compared with the OBPs, the CSP family displayed a simpler exon–intron organization but greater variation in total gene length. Motif 1 was shared across all PyouCSPs and thus represented the most conserved motif in this family. Each CSP contained the characteristic OS-D domain. The corresponding genes contained 2–4 exons and 1–3 introns. Although most CSP genes were shorter than 5 kb, PyouCSP7 was exceptionally long, spanning 32.1 kb. Except for PyouCSP2, all CSP genes contained at least one UTR, and PyouCSP7 showed the most complex UTR structure (Figure 1D).

The membrane receptor families exhibited more pronounced heterogeneity in motif composition and gene structure. In the OR family, a conserved 3-2-1 motif arrangement was observed in all members except PyouOR7, and all proteins contained the diagnostic 7tm_6 domain. OR genes comprised 4–15 exons and 3–14 introns, with considerable variation in genomic span. Most members were shorter than 5 kb, whereas PyouOR7 extended to 20.3 kb. Only PyouOR2, PyouOR6, and PyouOR7 possessed annotated UTRs (Figure 6A). In the GR family, motifs 1 and 2 were the most prevalent and occurred in every member. Notably, the bitter receptor clade containing PyouGR6–PyouGR10 and PyouGR12 exhibited a characteristic motif arrangement of 3-4-2-6-1. All PyouGRs harbored the conserved 7tm_7 domain. Their gene structures consisted of 4–10 exons and 4–10 introns, and most genes were shorter than 5 kb, although PyouGR14 reached 12.2 kb. Among all GR genes, only PyouGR2 retained both 5′ and 3′ UTRs (Figure 6B).

The IR family showed the highest level of structural complexity. PyouIR25a and PyouIR8a shared an identical motif arrangement of 8-4-7-3-1-5-2-9, underscoring their conserved status as core IR co-receptors (Figure 7A). Across the family, motif 2 was present in all members except PyouIR1, whereas motif 9 was absent only from PyouIR76b. The overall conserved motif order in the family was 8-10-6-4-7-3-1-5-2-9. Most PyouIRs carried the Type2 PBP domain. Their exon–intron structures varied extensively, comprising 1–22 exons and 0–21 introns. Gene length ranged from 1.6 kb in PyouIR10 to 41.5 kb in PyouIR14, and more than half of the IR genes (56.5%) contained 10 or more exons. Approximately 21.7% of the IR genes retained both 5′ and 3′ UTRs, whereas 47.8% contained a single UTR. The sole SNMP gene, PyouSNMP2, possessed the complete motif set arranged as 9-4-6-7-10-5-2-1-8-3 and contained the characteristic CD36 domain (Figure 7B). This gene consisted of 10 exons and 9 introns, spanned 21.3 kb, and lacked annotated UTR sequences.

### 3.7. Chromosomal Localization and Collinearity of Chemosensory Genes

**Figure 8:**
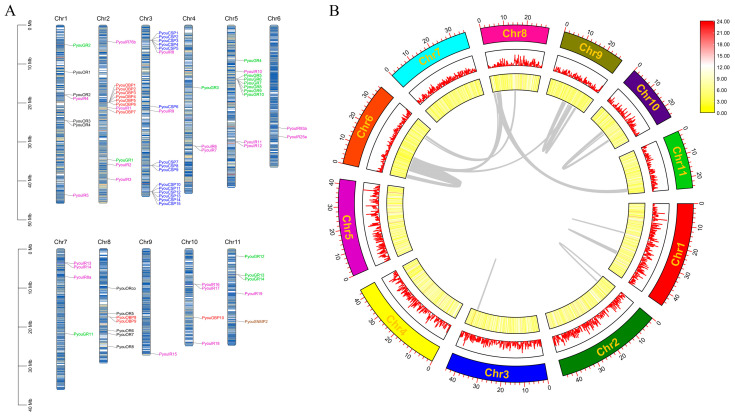
Chromosomal localization and collinearity of all chemosensory genes in *P. youi*. (**A**) Gene localization and tandem arrangement. Red represents OBPs, blue represents CSPs, black represents ORs, green represents GRs, purple represents IRs, and brown represents SNMPs. Inner chromosomal lines represent gene density. (**B**) Gene density and collinearity. Gray lines indicate segmental duplication events between genes. Chromosome numbers are labeled in yellow inside the outermost box. Gene density is shown in the two inner boxes as a line plot and a heatmap, respectively.

According to the genome annotation, all 72 chemosensory genes identified in *P. youi* could be anchored to 11 chromosomes. Their chromosomal distribution was clearly uneven (Figure 8A). Chromosome 3 contained the largest number of chemosensory genes (17 genes), whereas chromosome 9 harbored only a single member.

Distinct distributional patterns were evident among gene families. The 10 OBP genes were distributed across chromosomes 2, 4, and 8, with seven members concentrated on chromosome 2. In contrast, all 15 CSP genes were mapped to chromosome 3, where they formed an evident tandem cluster. The nine OR genes were located on chromosomes 1 and 8. The 14 GR genes were spread across chromosomes 1, 2, 4, 5, 7, and 11, with chromosome 7 containing the highest number of GRs (seven genes). Compared with the other families, IR genes displayed the broadest genomic distribution, being present on all chromosomes except chromosome 8. The single SNMP gene, PyouSNMP2, was located on chromosome 11 (Figure 8A).

At the chromosomal scale, chemosensory genes did not exhibit an obvious tendency to accumulate near either chromosomal end (Figure 8A). A total of 16 pairs of tandem duplicated chemosensory genes were identified in *P. youi*, including PyouOBP5 and PyouOBP6, PyouOBP8 and PyouOBP9, PyouCSP1 and PyouCSP2, PyouCSP3 and PyouCSP4, PyouCSP4 and PyouCSP5, PyouCSP7 and PyouCSP8, PyouCSP10 and PyouCSP11, PyouCSP11 and PyouCSP12, PyouGR6 and PyouGR7, PyouGR7 and PyouGR8, PyouGR8 and PyouGR9, PyouGR9 and PyouGR10, PyouGR13 and PyouGR14, PyouIR6 and PyouIR7, PyouIR13 and PyouIR14, and PyouIR16 and PyouIR17. Whole-genome collinearity analysis further failed to detect clear collinear relationships among members of the chemosensory gene families (Figure 8B), indicating that large-scale segmental duplication was not a prominent feature shaping the present chemosensory gene repertoire in *P. youi*.

## 4. Discussion

### 4.1. Overall Characteristics of the Chemosensory Repertoire in P. youi

Chemosensation constitutes a fundamental interface through which insects perceive and respond to their external environment, and comparative analyses of chemosensory gene families provide important insights into both sensory adaptation and the evolutionary diversification of insect lineages [63]. As a representative of Ephemeroptera, one of the earliest-diverging groups of winged insects, *P. youi* occupies a phylogenetically informative position for exploring the origin and evolutionary organization of insect chemosensory systems [64]. However, despite the ecological importance of mayflies, particularly their well-recognized sensitivity to freshwater environments, genome-wide investigations of their chemosensory gene repertoire remain scarce.

In the present study, we identified 72 putative chemosensory genes in *P. youi*, including 10 OBPs, 15 CSPs, 9 ORs, 14 GRs, 23 IRs, and 1 SNMP. Compared with the repertoires reported from many terrestrial insects, this total number is relatively small. For instance, more than 206 and 139 chemosensory genes have been reported in *L. migratoria* and *O. chinensis*, respectively [65,66,67], whereas several beetles, including *M. signata*, *P. brevitarsis*, and *P. yasumatsui*, possess 113–117 members [68,69,70]. Similarly, the lepidopteran species *Cnaphalocrocis medinalis* and *H. armigera* harbor 131 and 133 chemosensory genes, respectively [71,72]. Against this background, the repertoire identified in *P. youi* can be regarded as comparatively compact. However, direct comparisons across studies should be interpreted cautiously because the referenced datasets differ in sequencing source, assembly quality, gene annotation strategy, and completeness of chemosensory gene curation.

This comparatively small repertoire may be associated with the ecological context of mayflies, particularly their aquatic nymphal stage. In contrast to terrestrial insects that often cope with chemically complex airborne and plant-associated environments, mayfly nymphs are primarily exposed to water-borne signals related to algae, detritus, humic material, and suspended particles [64,73,74]. Such ecological conditions may impose a narrower range of chemically relevant cues and may reduce selective pressure for extensive expansion of some peripheral chemosensory gene families [75]. Nevertheless, this interpretation remains inferential. The relatively small number of chemosensory genes detected in *P. youi* may also be influenced by technical factors, including genome assembly limitations, incomplete capture of repetitive regions, fragmented gene models, or incomplete annotation. These limitations could lead to underestimation of the actual size of some gene families. Thus, the compact chemosensory repertoire observed here should be regarded as a cautious comparative inference rather than a definitive demonstration of broad repertoire reduction. Given the prolonged aquatic nymphal stage and short-lived adult stage of mayflies, potential differences in chemosensory demands across life stages may also have contributed to the repertoire pattern observed in *P. youi*, which warrants further investigation.

### 4.2. Family-Specific Patterns in Soluble Binding Proteins and Membrane Receptor Repertoires

The soluble binding protein families of *P. youi*, namely OBPs and CSPs, exhibit both conserved structural features and distinct patterns of family size. As the first molecular components involved in peripheral chemoreception, OBPs and CSPs are generally considered to facilitate the transport of hydrophobic ligands through the sensillum lymph to receptor proteins [76]. In *P. youi*, only 10 OBPs were identified, a number substantially lower than those reported in several terrestrial insects, including *L. migratoria*, *O. chinensis*, *S. gregaria*, *H. armigera*, *P. brevitarsis*, and *M. signata* [65,68,76,77,78,79]. By contrast, the 15-member CSP family does not appear exceptionally reduced and falls within the range commonly observed in insects. This asymmetry suggests that different soluble chemosensory-related protein families may have experienced distinct evolutionary constraints in *P. youi*.

The structural features of these proteins indicate substantial functional conservation. Most PyouOBPs and PyouCSPs possess predicted signal peptides, are likely secreted into extracellular space, and retain the characteristic cysteine frameworks typical of their respective families (Tables S4 and S5). Within the OBP family, the predominance of Classic OBPs is consistent with patterns observed across multiple insect orders, suggesting that this ancestral OBP type remains the principal soluble odorant-binding form in *P. youi*. Meanwhile, the close phylogenetic affinity between PyouOBP3/PyouOBP4 and PyouOBP8/PyouOBP9 may reflect lineage-specific duplication or local diversification. For CSPs, the retention of the canonical four-cysteine motif, together with the chromosomal clustering of all 15 members, suggests a family that is structurally conserved yet genomically concentrated. Given that CSPs are frequently implicated not only in chemoreception but also in broader physiological processes such as development and chemical communication, the maintenance of a moderate CSP repertoire in *P. youi* may reflect functional versatility rather than exclusively olfactory demand.

In contrast to the soluble protein families, the membrane receptor repertoires show a more uneven pattern of retention. The OR family, which represents the principal receptor system for volatile odorants in insects, is particularly compact in *P. youi*. Only nine ORs were identified, including a single ORco. Because ORco functions as a highly conserved co-receptor essential for receptor trafficking and signaling [80,81], its presence was expected. The small number of conventional ORs suggests that the canonical OR-based olfactory system of *P. youi* may be limited in scale relative to those of many Orthoptera, Coleoptera, and Lepidoptera, although this comparison should be interpreted cautiously because of differences in data sources and annotation strategies among studies. Phylogenetically, PyouORco remained highly conserved, whereas the conventional ORs formed two *P. youi*-specific clusters, suggesting that limited lineage-specific differentiation has occurred despite the small repertoire size. A similarly selective retention pattern was observed in the GR family. GRs are mainly involved in the perception of non-volatile compounds, including sugars, deterrents, and other contact-mediated chemical cues [82,83]. In *P. youi*, 14 GRs were identified, and these were restricted to the bitter receptor and sugar receptor lineages. No homologs corresponding to the CO_2_ receptor or fructose receptor subfamilies were detected, although this apparent absence should be interpreted cautiously. The apparent absence of detectable CO_2_-GR homologs in *P. youi* may reflect lineage-specific loss associated with its aquatic nymphal stage and short-lived adult stage, but it may also result from genome annotation limitations or high sequence divergence. Future tissue- and stage-specific transcriptomic analyses will be needed to verify this result and clarify how developmental stage, habitat, and feeding ecology shape the chemosensory repertoire of *P. youi*. This restricted composition suggests that the GR repertoire of *P. youi* has not diversified broadly but may have retained receptor types most relevant to its ecological context. Because *P. youi* is mainly associated with algae, detrital material, and fine particulate organic matter [36], receptors involved in nutrient evaluation and the detection of potentially harmful compounds may be particularly relevant. Notably, most PyouGRs were assigned to bitter receptor clades, suggesting that the bitter receptor lineage has been the principal axis of diversification within this family.

By contrast, the IR family remains comparatively well represented in *P. youi*. IRs are evolutionarily derived from iGluRs and constitute an ancient chemosensory system distinct from the OR and GR superfamilies [84]. We identified 23 IRs, including the four conserved co-receptors IR8a, IR25a, IR76b, and IR93a. The retention of all major co-receptors, together with multiple divergent IRs, suggests that IR-mediated sensory pathways may remain functionally important in *P. youi*. This inference is supported by the phylogenetic placement of PyouIRs into conserved clades such as IR21a and IR40a, as well as into NMDA-like, non-NMDA-like, and divergent IR lineages, suggesting that multiple ancestral IR-associated sensory functions may have been preserved. The comparatively rich IR repertoire may be ecologically meaningful. In other insects, IRs participate in the detection of acids, amines, humidity, temperature-related cues, and other environmental signals [85,86,87,88,89]. For an aquatic or semi-aquatic lineage such as mayflies, these modalities may be especially relevant because water-borne chemical gradients and physicochemical environmental parameters are likely to constitute major components of the sensory landscape. It is therefore plausible that *P. youi* relies relatively more on IR-mediated detection of dissolved or environmental cues than on extensive OR-based discrimination of airborne odorants. However, because direct functional evidence is still lacking, this interpretation should be considered a hypothesis for future testing.

The SNMP family appears to be limited in *P. youi*. Only a single SNMP gene, PyouSNMP2, was identified, and phylogenetic analysis placed it within the SNMP2 clade [90]. The encoded protein retained the typical two-transmembrane architecture characteristic of CD36 family members, indicating structural conservation despite the low copy number. In many insects, SNMPs are involved in pheromone or lipid-like ligand detection and may facilitate OR-dependent chemosensory signaling [91,92]. In this context, the absence of a detectable SNMP1 homolog, together with the small OR repertoire, may suggest a simplified SNMP-associated olfactory pathway in *P. youi*. This interpretation, however, should be approached cautiously, as apparent gene absence in a genome annotation dataset may also reflect assembly or annotation limitations. Moreover, because several receptor models showed atypical lengths or transmembrane-domain numbers, annotation-related uncertainty cannot be completely excluded, and future transcriptomic validation will be necessary to further confirm these loci.

### 4.3. Structural Organization and Genomic Distribution of Chemosensory Genes

The analyses of conserved motifs, domain organization, and exon–intron structure revealed substantial differences among chemosensory gene families in *P. youi*. OBPs and CSPs displayed relatively stable motif composition and comparatively conserved gene structures, consistent with the expectation that soluble carrier proteins are subject to stronger structural constraints. By contrast, ORs and GRs showed greater heterogeneity in both motif arrangement and exon–intron organization, indicating a higher degree of architectural diversification among membrane receptor genes. The IR family exhibited the broadest variation in gene length, exon number, and UTR composition, suggesting that different IR members have followed markedly divergent structural trajectories during evolution [93,94,95,96,97]. Importantly, such structural complexity should not be interpreted simply as evidence of recent expansion; rather, it may reflect the deep evolutionary origin and broad functional scope of the IR family.

Chromosomal localization analysis provided additional information on the genomic organization of chemosensory genes in *P. youi*. The 72 genes were unevenly distributed across all 11 chromosomes, and several families showed clear concentration patterns, including the complete clustering of CSPs on chromosome 3, the enrichment of OBPs on chromosome 2, and the accumulation of multiple GRs on chromosome 7. In insect genomes, such clustered distributions are often associated with tandem duplication or local retention following duplication events [98]. Combined with tandem duplication analysis, these distributional patterns indicate that tandem gene duplication occurred within the CSP, OBP, and GR families. The family-specific concentration patterns observed here are therefore compatible with localized expansion processes, especially for the CSP and GR families. By contrast, whole-genome collinearity analysis did not reveal obvious collinear relationships among the chemosensory genes. This result suggests that large-scale segmental duplication has not been a major force in shaping the current chemosensory repertoire in *P. youi*. It should be noted, however, that the absence of clear collinearity does not by itself provide direct evidence for chromosomal rearrangement. A more conservative interpretation is that the present gene complement is more likely to reflect localized family-specific organization, together with selective retention and gene loss, than extensive segmental duplication. Comparative genomic analyses involving additional mayfly species will be necessary to clarify the respective contributions of tandem duplication, lineage-specific reduction, and long-term chromosomal evolution to chemosensory gene diversification in Ephemeroptera.

Overall, the chemosensory repertoire of *P. youi* appears to be characterized by a comparatively small size together with clear family-specific differences in evolutionary pattern. In particular, the relatively rich IR repertoire, contrasted with the small OR and SNMP complements, suggests that different chemosensory pathways may contribute unequally to sensory adaptation in this species. These findings provide a useful framework for future functional and comparative studies of chemosensation in mayflies.

## 5. Conclusions

We identified 72 putative chemosensory genes in *P. youi*, including members of the OBP, CSP, OR, GR, IR, and SNMP families, and characterized their phylogenetic relationships, structural features, and chromosomal distribution at the genome-wide level. The results reveal a compact but non-uniform chemosensory repertoire, with marked family-specific differences in evolutionary pattern and genomic organization. In particular, the comparatively rich IR complement, contrasted with the small OR and SNMP repertoires, highlights potential differences in the contribution of distinct chemosensory pathways to sensory adaptation in *P. youi*. These findings provide a genomic basis for future functional and comparative studies of chemosensation in mayflies.

## Data Availability

The original contributions presented in this study are included in the article and supplementary material. Further inquiries can be directed to the corresponding author(s).

## Supplementary Materials

The following supporting information can be downloaded at: https://www.mdpi.com/article/10.3390/genes17050549/s1, Table S1: Nucleotide sequences of identified chemosensory genes; Table S2: Amino acid sequences of identified chemosensory genes; Table S3: Species color-coding table for phylogenetic analysis; Table S4: Summary of putative odorant binding proteins (OBPs) identified in *P. youi*; Table S5: Summary of putative chemosensory proteins (CSPs) identified in *P. youi*; Table S6: Summary of putative odorant receptors (ORs) identified in *P. youi*; Table S7: Summary of putative gustatory receptors (GRs) identified in *P. youi*; Table S8: Summary of putative ionotropic receptors (IRs) and sensory neuron membrane proteins (SNMPs) identified in *P. youi*.
