# Supplementary material for: Genome-Wide Identification and Characterization of Chemosensory Gene Families in the Mayfly Parafronurus youi (Ephemeroptera: Heptageniidae)
Source: Genes (Basel). 2026 May 4;17(5):549. doi: 10.3390/genes17050549 (PMC13205551; doi:10.3390/genes17050549)
Supplement: Supplementary file 1 [file genes-17-00549-s001.zip › Table S3.pdf]

Table S3. Species color-coding table for phylogenetic analysis.

Gene family	Species	Colors
OBP	*Parafronurus youi*	Red
	Locusta migratoria	DarkOrange
	Oxya chinensis	MediumBlue
	Helicoverpa armigera	LimeGreen
	Schistocerca gregaria	Magenta
	Protaetia brevitarsis	SkyBlue
	Monolepta signata	Brown
CSP	*Parafronurus youi*	Red
	Tribolium castaneum	MediumBlue
	Protaetia brevitarsis	RoyalBlue
	Pachyrhinus yasumatsui	DarkViolet
	Monolepta signata	SeaGreen
	Helicoverpa armigera	LimeGreen
	Bombyx mori	Magenta
	Oxya chinensis	DarkOrange
	Locusta migratoria	SkyBlue
	Ceracris kiangsu	SlateGrey
	Ceracris nigricornis	Brown
OR	*Parafronurus youi*	Red
	Locusta migratoria	DarkViolet
	Oxya chinensis	LimeGreen
GR	*Parafronurus youi*	Red
	Pachyrhinus yasumatsui	DarkViolet
	Monolepta signata	Magenta
	Dendroctonus ponderosae	MediumBlue
	Agrilus planipennis	SkyBlue
	Sympiezomias velatus	LimeGreen
IR	*Parafronurus youi*	Red
	Locusta migratoria	skyblue
	Oxya chinensis	DarkViolet
	Monolepta signata	LimeGreen
	Pachyrhinus yasumatsui	DarkOrange
	Bombyx mori	Magenta
SNMP	*Parafronurus youi*	Red
	Monolepta signata	BlueViolet
	Pachyrhinus yasumatsui	LimeGreen
	Oxya chinensis	Plum
	Schistocerca gregaria	MediumBlue
	Oedaleus asiaticus	SkyBlue
	Ceracris kiangsu	Magenta
	Aedes aegypti	SlateGrey
	Apis mellifera	DodgerBlue

**Gene family**	**Species**	**Colors**
	Bombyx mori	Salmon
	Drosophila melanogaster	Gold
	Tribolium castaneum	blue
	Dendroctonus ponderosae	Brown
SNMP	*Anoplophora glabripennis*	DarkOrange
	Curculio dieckamanni	DeepPink
	Callosobruchus maculatus	Violet
	Plagiodera versicolora	green
	Adelphocoris lineolatus	RoyalBlue
