# Supplementary material for: Genome-Wide Identification and Characterization of Chemosensory Gene Families in the Mayfly Parafronurus youi (Ephemeroptera: Heptageniidae)
Source: Genes (Basel). 2026 May 4;17(5):549. doi: 10.3390/genes17050549 (PMC13205551; doi:10.3390/genes17050549)
Supplement: Supplementary file 1 [file genes-17-00549-s001.zip › Table S4.pdf]

Table S4. Summary of putative odorant binding proteins (OBPs) identified in *P. youi*.

Gene Name	Gene ID	Length (nt)	ORF (aa)	Signal Peptide	Molecular Weight (kD)	Isoelectric Point	Instability Index	Aliphatic Index	Grand Average of Hydropathicity	Subcellular Localization
PyouOBP1	Parafronurus_youi_00000671-RA	465	154	1-24	17.31	5.04	37.58	84.22	-0.091	extr
PyouOBP2	Parafronurus_youi_00000672-RB	450	149	1-18	17.00	5.64	25.13	86.38	0.036	extr
PyouOBP3	Parafronurus_youi_00000674-RA	447	148	1-20	16.86	4.69	54.08	75.14	-0.433	extr
PyouOBP4	Parafronurus_youi_00000675-RA	450	149	1-20	17.07	6.12	25.22	83.76	-0.232	extr
PyouOBP5	Parafronurus_youi_00000677-RA	408	135	1-21	15.00	8.69	12.52	83.85	-0.359	extr
PyouOBP6	Parafronurus_youi_00000678-RA	423	140	1-21	15.40	8.40	33.36	94.14	-0.025	extr
PyouOBP7	Parafronurus_youi_00000822-RA	495	164	1-22	19.25	5.58	39.96	83.23	-0.454	extr
PyouOBP8	Parafronurus_youi_00011250-RA	426	141	1-19	15.65	6.28	41.97	78.23	-0.179	extr
PyouOBP9	Parafronurus_youi_00011251-RA	432	143	1-19	15.69	6.55	36.58	81.26	-0.049	extr
PyouOBP10	Parafronurus_youi_00014260-RA	759	252	1-20	27.53	9.50	56.72	65.91	-0.597	extr
