# Supplementary material for: Genome-Wide Identification and Characterization of Chemosensory Gene Families in the Mayfly Parafronurus youi (Ephemeroptera: Heptageniidae)
Source: Genes (Basel). 2026 May 4;17(5):549. doi: 10.3390/genes17050549 (PMC13205551; doi:10.3390/genes17050549)
Supplement: Supplementary file 1 [file genes-17-00549-s001.zip › Table S5.pdf]

Table S5. Summary of putative chemosensory proteins (CSPs) identified in *P. youi.*

Gene Name	Gene ID	Length (nt)	ORF (aa)	Signal Peptide	Molecular Weight (kD)	Isoelectric Point	Instability Index	Aliphatic Index	Grand Average of Hydropathicity	Subcellular Localization
PyouCSP1	Parafronurus_youi_00006008-RA	369	122	1-17	13.85	8.25	30.19	95.90	-0.129	extr
PyouCSP2	Parafronurus_youi_00006009-RA	399	132	1-20	14.97	9.03	51.76	83.56	-0.455	extr
PyouCSP3	Parafronurus_youi_00006011-RA	381	126	1-20	14.51	8.29	42.49	89.05	-0.526	extr
PyouCSP4	Parafronurus_youi_00006012-RA	360	119	1-19	13.61	6.70	32.51	82.86	-0.471	extr
PyouCSP5	Parafronurus_youi_00006013-RA	399	132	1-17	15.03	6.56	41.70	81.29	-0.426	extr
PyouCSP6	Parafronurus_youi_00006670-RA	414	137	1-23	14.77	9.20	52.61	83.50	-0.076	extr
PyouCSP7	Parafronurus_youi_00007254-RA	654	217	No	24.24	8.31	61.61	71.98	-0.429	extr
PyouCSP8	Parafronurus_youi_00007255-RA	390	129	1-19	14.51	9.41	33.60	82.48	-0.481	extr
PyouCSP9	Parafronurus_youi_00007256-RA	303	100	1-19	11.37	9.78	38.50	97.60	-0.217	extr
PyouCSP10	Parafronurus_youi_00007513-RA	630	209	No	23.46	7.64	64.85	77.03	-0.454	extr
PyouCSP11	Parafronurus_youi_00007514-RA	396	131	1-18	14.74	8.33	57.24	86.41	-0.504	extr

Table S5. Summary of putative chemosensory proteins (CSPs) identified in *P. youi*.

Gene Name	Gene ID	Length (nt)	ORF (aa)	Signal Peptide	Molecular Weight (kD)	Isoelectric Point	Instability Index	Aliphatic Index	Grand Average of Hydropathicity	Subcellular Localization
PyouCSP12	Parafronurus_youi_00007515-RA	624	207	No	22.60	5.29	62.50	69.47	-0.665	extr
PyouCSP13	Parafronurus_youi_00007516-RA	387	128	1-17	13.97	6.81	50.29	85.39	-0.120	extr
PyouCSP14	Parafronurus_youi_00007517-RA	336	111	1-17	11.88	7.65	24.40	98.65	0.033	extr
PyouCSP15	Parafronurus_youi_00007518-RA	414	137	1-18	15.94	8.17	69.11	86.06	-0.499	extr
