# Supplementary material for: Genome-Wide Identification and Characterization of Chemosensory Gene Families in the Mayfly Parafronurus youi (Ephemeroptera: Heptageniidae)
Source: Genes (Basel). 2026 May 4;17(5):549. doi: 10.3390/genes17050549 (PMC13205551; doi:10.3390/genes17050549)
Supplement: Supplementary file 1 [file genes-17-00549-s001.zip › Table S6.pdf]

Table S6. Summary of putative odorant receptors (ORs) identified in *P. youi.*

Gene Name	Gene ID	Length (nt)	ORF (aa)	Tm domain	Molecular Weight (kD)	Isoelectric Point	Instability Index	Aliphatic Index	Grand Average of Hydropathicity	Subcellular Localization
PyouOR1	Parafronurus_youi_ 00002493-RA	624	207	2	22.95	8.47	29.30	104.06	0.414	cyto
PyouOR2	Parafronurus_youi_ 00002719-RA	1509	502	8	56.93	9.02	29.17	113.98	0.661	plas
PyouOR3	Parafronurus_youi_ 00003022-RA	567	188	3	21.28	5.01	35.55	109.41	0.432	plas
PyouOR4	Parafronurus_youi_ 00003024-RA	453	150	3	16.66	8.72	29.51	129.33	0.871	plas
PyouOR5	Parafronurus_youi_ 00011222-RA	1062	353	5	39.53	8.62	30.16	104.76	0.389	plas
PyouOR6	Parafronurus_youi_ 00011399-RA	918	305	4	33.49	9.12	37.55	108.66	0.549	plas
PyouOR7	Parafronurus_youi_ 00011443-RA	2598	865	5	99.34	6.04	46.27	95.72	0.008	plas
PyouOR8	Parafronurus_youi_ 00011544-RA	708	235	4	26.93	7.70	42.14	112.00	0.478	plas
PyouORco	Parafronurus_youi_ 00010970-RA	1293	430	5	48.65	5.51	32.14	100.14	0.220	plas
