# Supplementary material for: Genome-Wide Identification and Characterization of Chemosensory Gene Families in the Mayfly Parafronurus youi (Ephemeroptera: Heptageniidae)
Source: Genes (Basel). 2026 May 4;17(5):549. doi: 10.3390/genes17050549 (PMC13205551; doi:10.3390/genes17050549)
Supplement: Supplementary file 1 [file genes-17-00549-s001.zip › Table S7.pdf]

Table S7. Summary of putative gustatory receptors (GRs) identified in *P. youi.*

Gene Name	Gene ID	Length (nt)	ORF (aa)	Tm domain	Molecular Weight (kD)	Isoelectric Point	Instability Index	Aliphatic Index	Grand Average of Hydropathicity	Subcellular Localization
PyouGR1	Parafronurus_youi_00001391-RA	1536	511	7	56.11	8.31	35.67	100.59	0.251	plas
PyouGR2	Parafronurus_youi_00002206-RA	1236	411	7	46.94	8.49	36.02	111.97	0.441	plas
PyouGR3	Parafronurus_youi_00004646-RA	1233	410	7	45.29	8.04	37.49	117.78	0.703	plas
PyouGR4	Parafronurus_youi_00008025-RA	1269	422	6	48.10	8.82	38.55	111.64	0.297	plas
PyouGR5	Parafronurus_youi_00008229-RA	1104	367	6	40.79	8.81	47.19	118.99	0.429	plas
PyouGR6	Parafronurus_youi_00008243-RA	1215	404	7	46.81	6.00	33.72	119.43	0.515	plas
PyouGR7	Parafronurus_youi_00008244-RA	1239	412	7	47.57	9.33	34.58	118.98	0.496	plas
PyouGR8	Parafronurus_youi_00008245-RA	1212	403	6	46.35	6.19	39.46	122.18	0.571	plas
PyouGR9	Parafronurus_youi_00008246-RA	1026	341	6	39.13	7.12	36.59	113.52	0.390	plas
PyouGR10	Parafronurus_youi_00008247-RA	1200	399	7	45.41	8.93	37.17	117.82	0.481	plas
PyouGR11	Parafronurus_youi_00010231-RA	1236	411	2	46.33	9.54	45.17	116.23	0.427	plas

Table S7. Summary of putative gustatory receptors (GRs) identified in *P. youi*.

Gene Name	Gene ID	Length (nt)	ORF (aa)	Tm domain	Molecular Weight (kD)	Isoelectric Point	Instability Index	Aliphatic Index	Grand Average of Hydropathicity	Subcellular Localization
PyouGR12	Parafronurus_youi_ 00014551-RA	1218	405	7	46.55	8.59	39.83	120.15	0.488	plas
PyouGR13	Parafronurus_youi_ 00014688-RA	1248	415	0	48.67	4.55	55.72	89.95	-0.448	nucl
PyouGR14	Parafronurus_youi_ 00014689-RA	1185	394	0	46.05	6.05	53.50	111.80	0.194	plas
