# Supplementary material for: Genome-Wide Identification and Characterization of Chemosensory Gene Families in the Mayfly Parafronurus youi (Ephemeroptera: Heptageniidae)
Source: Genes (Basel). 2026 May 4;17(5):549. doi: 10.3390/genes17050549 (PMC13205551; doi:10.3390/genes17050549)
Supplement: Supplementary file 1 [file genes-17-00549-s001.zip › Table S8.pdf]

Table S8. Summary of putative ionotropic receptors (IRs) and sensory neuron membrane protein (SNMP) identified in *P. youi.*

Gene Name	Gene ID	Length (nt)	ORF (aa)	Tm domain	Molecular Weight (kD)	Isoelectric Point	Instability Index	Aliphatic Index	Grand Average of Hydropathicity	Subcellular Localization
PyouIR1	Parafronurus_youi_00000760-RA	2268	755	2	83.92	8.56	56.32	90.15	-0.109	plas
PyouIR2	Parafronurus_youi_00001469-RA	2001	666	1	74.94	9.86	52.14	105.56	0.032	nucl
PyouIR3	Parafronurus_youi_00001679-RA	1740	579	2	65.20	6.37	43.77	109.46	0.167	plas
PyouIR4	Parafronurus_youi_00002736-RA	1002	333	1	38.04	9.85	45.39	100.75	-0.121	plas
PyouIR5	Parafronurus_youi_00003926-RA	852	283	1	31.95	9.53	35.97	99.19	0.166	plas
PyouIR6	Parafronurus_youi_00005308-RA	2004	667	3	75.89	8.99	45.93	89.61	-0.099	E.R
PyouIR7	Parafronurus_youi_00005309-RA	1851	616	2	69.03	8.88	49.89	87.58	-0.139	nucl
PyouIR8	Parafronurus_youi_00006019-RA	867	288	1	32.13	6.51	35.37	98.85	-0.139	pero
PyouIR9	Parafronurus_youi_00006716-RC	2679	892	3	100.21	5.89	43.65	86.46	-0.171	plas
PyouIR10	Parafronurus_youi_00008124-RB	1551	516	1	58.13	9.15	40.94	112.38	0.108	plas
PyouIR11	Parafronurus_youi_00008877-RA	2694	897	3	102.45	7.58	45.90	86.61	-0.244	plas

Table S8. Summary of putative ionotropic receptors (IRs) and sensory neuron membrane protein (SNMP) identified in *P. youi.*

Gene Name	Gene ID	Length (nt)	ORF (aa)	Tm domain	Molecular Weight (kD)	Isoelectric Point	Instability Index	Aliphatic Index	Grand Average of Hydropathicity	Subcellular Localization
PyouIR12	Parafronurus_youi_00008884-RA	3009	1002	3	113.70	8.02	33.03	90.02	-0.112	plas
PyouIR13	Parafronurus_youi_00009496-RA	2838	945	3	108.14	6.23	40.68	90.15	-0.208	plas
PyouIR14	Parafronurus_youi_00009497-RA	3180	1059	4	120.57	6.09	44.39	85.36	-0.168	plas
PyouIR15	Parafronurus_youi_00013634-RB	3483	1160	4	128.32	10.13	48.90	92.13	-0.109	plas
PyouIR16	Parafronurus_youi_00013934-RA	2718	905	4	101.72	5.57	46.05	87.81	-0.191	plas
PyouIR17	Parafronurus_youi_00013935-RB	2733	910	3	102.43	7.84	41.20	82.42	-0.257	plas
PyouIR18	Parafronurus_youi_00014431-RA	2886	961	4	106.94	5.48	35.42	90.29	-0.153	plas
PyouIR19	Parafronurus_youi_00014828-RA	2934	977	3	111.33	8.98	40.86	86.63	-0.224	plas
PyouIR8a	Parafronurus_youi_00009628-RA	2826	941	3	105.13	6.45	39.78	89.26	-0.116	plas
PyouIR25a	Parafronurus_youi_00012476-RA	2637	878	3	99.62	5.45	42.46	90.08	-0.208	plas
PyouIR76b	Parafronurus_youi_00000178-RA	1533	510	3	57.75	8.64	54.79	99.63	0.064	plas

Table S8. Summary of putative ionotropic receptors (IRs) and sensory neuron membrane protein (SNMP) identified in *P. youi.*

Gene Name	Gene ID	Length (nt)	ORF (aa)	Tm domain	Molecular Weight (kD)	Isoelectric Point	Instability Index	Aliphatic Index	Grand Average of Hydropathicity	Subcellular Localization
PyouIR93a	Parafronurus_youi_00012417-RA	2421	806	4	90.46	6.50	44.77	95.71	-0.016	plas
PyouSNMP2	Parafronurus_youi_00015058-RA	1587	528	2	58.04	4.61	30.77	101.34	0.069	plas
